# Phytochemistry, Food Application, and Therapeutic Potential of the Medicinal Plant (*Withania coagulans*): A Review

**DOI:** 10.3390/molecules26226881

**Published:** 2021-11-15

**Authors:** Muhammad Issa Khan, Maria Maqsood, Raakia Anam Saeed, Amna Alam, Amna Sahar, Marek Kieliszek, Antoni Miecznikowski, Hafiz Shehzad Muzammil, Rana Muhammad Aadil

**Affiliations:** 1 National Institute of Food Science and Technology, University of Agriculture, Faisalabad 38000, Pakistan; mariamaqsood75@gmail.com (M.M.); raakia.anam@gmail.com (R.A.S.); amnahnd17@gmail.com (A.A.); amnasahar@uaf.edu.pk (A.S.); shehzad.muzammil@uaf.edu.pk (H.S.M.); 2 Department of Food Engineering, University of Agriculture, Faisalabad 38000, Pakistan; 3 Department of Food Biotechnology and Microbiology, Institute of Food Sciences, Warsaw University of Life Sciences—SGGW, Nowoursynowska 159 C, 02-776 Warsaw, Poland; marek_kieliszek@sggw.edu.pl; 4 Department of Fermentation Technology, Prof. Waclaw Dabrowski Institute of Agricultural and Food Biotechnology—State Research Institute, Rakowiecka 36, 02-532 Warsaw, Poland; antoni.miecznikowski@ibprs.pl

**Keywords:** Ayurvedic properties, diuretic, phytochemistry, pharmacognostic properties

## Abstract

Herbal plants have been utilized to treat and cure various health-related problems since ancient times. The use of Ayurvedic medicine is very significant because of its least reported side effects and host of advantages. *Withania coagulans* (Family; Solanaceae), a valuable medicinal plant, has been used to cure abnormal cell growth, wasting disorders, neural as well as physical problems, diabetes mellitus, insomnia, acute and chronic hepatic ailments. This review provides critical insight regarding the phytochemistry, biological activities, and pharmacognostic properties of *W. coagulans*. It has been known to possess diuretic, anti-inflammatory, anti-bacterial, anti-fungal, cardio-protective, hepato-protective, hypoglycemic, anti-oxidative, and anti-mutagenic properties owing to the existence of withanolides, an active compound present in it. Apart from withanolides, *W. coagulans* also contains many phytochemicals such as flavonoids, tannins, and β-sterols. Several studies indicate that various parts of *W. coagulans* and their active constituents have numerous pharmacological and therapeutic properties and thus can be considered as a new drug therapy against multiple diseases.

## 1. Introduction

**Figure 1:**
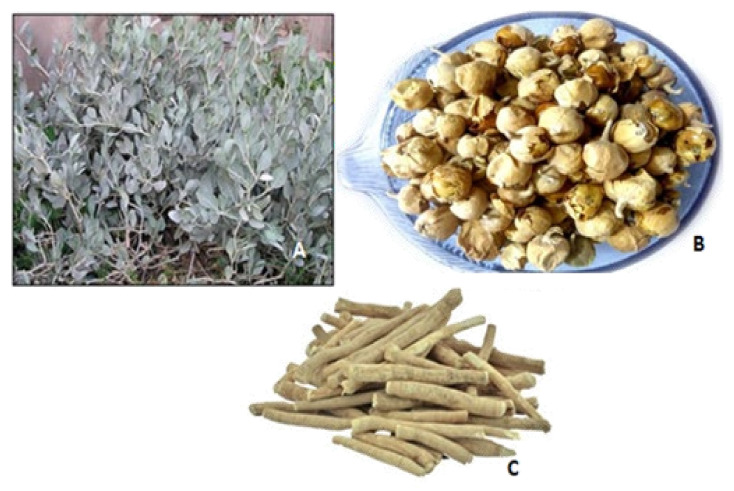
*W. coagulans* plant. (**A**): Leaves, (**B**): Fruits, (**C**): Stems.

Plants are potential sources of medicinal compounds and have been used as ancient healing systems since antiquity. Some medicinal plants are enriched with diverse bioactive constituents. These bioactive constituents have been reported as beneficial to prevent and treat various disorders for maintaining a healthy life. One of the most important medicinal plants of genus *Withania* is *Withania coagulans* in the Ayurvedic medicinal system owing to its effective nutraceutical as well as pharmaceutical attributes. It is grown in various regions of the world such as in the Mediterranean region and from North Africa to South Asia [1]. Among twenty-three identified species of genus *Withania*, only two (*W. coagulans* and *W. somnifera*) have economic significance [2]. This plant is widely used to coagulate milk due to the presence of an enzyme in its berries, which is commonly known as an Indian cheesemaker [3]. Figure 1 depicts the leaves, stems, and fruit of the *W. coagulans* plant. The fruit, roots, and leaves have various therapeutic effects. The main constituents in berries include essential oils, esterases, amino acids, and alkaloids [4]. The healing properties of the plant are attributed to steroid derivative compounds “Withanolides”. There are several withanolides such as coagulin F, coagulanolide, withacoagulin, and coagulin G present in the whole plant [3]. The ripe fruit of the plant is sweet and used for wound healing, asthma and dyspepsia, and as a sedative. In many countries, dry fruit is also used as a traditional treatment for diabetes [5] and as an antibacterial [6], antimicrobial [7], hepatoprotective [8], hypolipidemic [9], antioxidant [10], anti-tumor [11], antidepressant [12], immunosuppressive [13], and anti-inflammatory agent [14]. Seeds are useful for reducing inflammation, as a diuretic, and also in curing ophthalmia, while flower buds showed anthelmintic activity [15,16,17]. Twigs of the plant are used for cleaning teeth, as toothache and blood purifier agents in South Asian regions [3]. By considering the growing utilization of medicinal plants and their application in various indigenous health systems, this review is intended to provide comprehensive knowledge on phytochemistry, food uses, and the therapeutic potential of *W. coagulans.*

## 2. Nutritional Profile

*W. coagulans* is a renowned herb due to its ethnopharmacological properties. It has been utilized as a herbal remedy and is widely distributed in Iran, Pakistan, Afghanistan, and East India. *W. coagulans* is a good source of macro and micronutrients. The mineral composition of *W. coagulans* is summarized in Table 1. It contains a small fraction of moisture, protein, fat, and fiber and is also a good source of carbohydrates. Studies also indicate a higher amount of magnesium (greater than *Alhagi maurorum*, *Berberis lyceum*, and *Tecomella undulate*), calcium (greater than *Dature alba*, *A. maurorum*, *Chenopodium album*, *B. lyceum*, *T. undulate)*, potassium (greater than *B. lyceum* and *T. undulate)*, and iron (greater than *D. alba*, *B. lyceum*, and *T. undulata*) in *W. coagulans* [4]. Roots are composed of ash (1.92%), carbohydrates (75.71%), lipids (5.5%), protein (2.95%), and fiber (5.76%). Leaves are composed of ash (3.26%), carbohydrates (65.31%), lipids (5%), protein (2.95%), and fiber (11.76%). Moreover, fruit contains ash (4.21%), carbohydrates (60.14%), lipids (5%), and protein (4.65%) [18].

The berries of the *W. coagulans* are composed of milk coagulating enzymes, two esterase, free amino acids, and essential oil. Proline, tyrosine, valine, hydroxyproline, glycine, cysteine, asparagine, glutamic, and aspartic acids are the main amino acids that are present in the plant. Major fatty acids include arachidonic acid, stearic acid, palmitic acid, linoleic and oleic acids [3]. Furthermore, *n*-octatriacont-17-enoic acid, geranilan-10-olyl dihydrocinnamoate (aromatic ester), and geranilan-8-oic acid-10-olyl salicyloxy-2-*O*-β-d-glucofuranosyl-(6″→1‴)-*O*-β-d-glucofuranosyl-6‴-*n*-octadec-9‴′,11‴′″-dienoate (monoterpenic benzyl glucoside) in consort with two already identified fatty acids named as *n*-dotriacont-21-enoic acid and *n*-tetratriacontanoic acid were characterized in berries [19]. Similarly, research also detected twenty constituents in the essential oil of the *W. coagulans* fruit including sesquiterpenes (54%) and esters (21.50%) as dominant compounds followed by the presence of fatty acids (5.5%) such as nonanoic acid, hexanoic acid, methyl ester of hexadecanoic acid, methyl ester of nondecanoic acid, methyl esters of 8,11-octadecadienoic acid, methyl ester of 9-octadecenoic acid, and ethyl ester of linoleic acid, alkanes (9.11%), and the aldehydes (0.32%) in smaller percentage [20]. The un-saponifiable matter of the plant seed is composed of triacontane as well as β-sitosterol and dihydrostigmasterol [21]. In addition, it was reported that seeds are composed of approximately 12–14% of oil. The presence of free sugar (17.8%) in the form of d-galactose and _D_-arabinose (1:1) in a de-fatted meal of the *W. coagulans* seeds was also elucidated with maltose present in trace amounts [22]. Higher percentages of β-sitosterol, as well as linoleic acid, were also found and reported for the hypo-cholesterolemic effect of corn oil in combination with *W. coagulans* [15,23].

## 3. Phytochemistry

*Withania* species have been studied extensively by several researchers that subsequently led to the identification, characterization, and isolation of bioactive compounds in different parts of a plant. It includes several steroidal lactones, tannins, flavonoids, and alkaloids [24,25,26]. Ten new phytoconstituents were identified from air-dried *W. coagulans* fruit extracted with methanol and their structures were based on their chemical and spectral data [27]. Various constituents of *W. coagulans* were estimated in three different extracts namely, methanolic, hydroalcoholic, and chloroform. It was reported that total phenolic content (55.9 mg/g), total tannins (76.6 mg/g), total flavonoids (0.88 mg/g), and total flavanol (0.25 mg/g) were higher in the methanolic extract as compared to hydroalcoholic and chloroformic [28].

In a study conducted in Iran, the presence of flavonoids (5.70–6.50%), anthocyanins (4.51–9.51 µmol/g), and total phenolics (14.91–23.7 µg gallic acid equivalent (GEA)/mg D.W) were confirmed in *W. coagulans* [29]. The leaves of *W. coagulans* demonstrated the levels of total phenolics (58.21 mg GEA/g) and flavonoids (47 mg rutin equivalent (RE)/g), respectively [30]. The important chemical constituents of this medicinal plant are the withanolides, a series of polyhydroxy steroidal lactones, mainly present in the leaves as well as roots. They are composed of C-28 steroidal lactones based on the ergostane structure and the six or five-membered lactone ring is formed by oxidation of C-22 and C-26 [25]. Their concentrations vary from 0.001% to 0.5% of the dry weight [26,31,32,33]. Two major groups include withanolides containing the modified carbocyclic structure and withanolides containing the unmodified skeleton (including the regular β–oriented side chains and the unusual α-oriented side chains). They are classified into seven groups based on being derivatives of ergostane and include 5β,6β–epoxides; 6α,7α–epoxides; 5-enes; intermediate compounds; 5α,6α–epoxides; 6β,7β-epoxides, and phenolic withanolides [31,34].

So far, several withanolides, alkaloids, and sitoindosides (withanolide with glucose molecule at carbon 27) have been reported and isolated from *Withania* species [24,26,35,36,37,38,39,40,41,42,43,44,45,46]. One of the most salient features that withanolide-producing plants possess is to host an oxygen function in almost all positions of side chains or carbocyclic skeletons. Novel structural variants arise by modifications in either the side chain or in the carbocyclic skeleton [15].

## 4. Novel Isolated Compounds of *W. coagulans*

**Figure 2:**
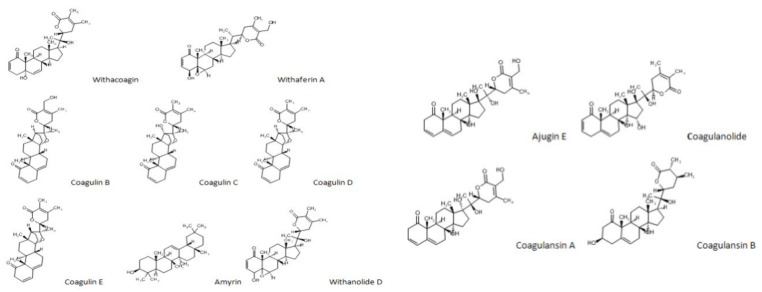
Structure of Withanolides of *W. Coagulans*.

Several compounds have been identified in different parts of *W. coagulans* including certain coagulans, coagulanolide, and coagulins. Choudhary et al., [40] identified 17β- hydroxywithanolide K: [(20*S*,22*R*)14α,17β,20β-trihydroxy-1-oxo-witha-2,5,24-trienolide] and 17β,20β-dihydroxy-1-oxo-witha-2,5,24-trienolide in whole plant. Similarly, Shahwar [47] found withahejarin, withasomniferine-A along with coagulin A. Furthermore, identification and isolation of thirteen coagulins (Coagulin F, G, H, I, J, K, L, M, N, O, P, Q, and R) from the whole plant was reported [41,48,49]. *W. coagulans* also contains coagulin U along with other metabolites such as methyl-4–benzoate and phytosterols (β-sitosterol, β-sitosterol glycoside). Similarly (22*R*),20β-hydroxy-1-oxowitha-2,5,24-trienolide and (22*R*)-14,20-epoxy-17β-hydroxy-1-oxowitha-3,5,25-trienolide which are also important constituents of it [50]. Moreover, 17β,27-dihydroxy-14,20-epoxy-1-oxo-22R-witha-3,5,24-trienolide and 17β-hydroxy-14α,20α-epoxy-1-oxo-(22*R*)-witha-3,5,24-trienolide were discovered [41]. Coagulin S was also isolated, and its structure was elucidated by using spectroscopic techniques [44]. Coagulansin B and coagulanolide are also amongst the metabolites of *W. coagulans* [51,52]. Withacoagulin J was identified as well as isolated along with already known withanolide H [53]. Withanolide named as (20*R*,22*R*)-14α,17,20β,27-trihydroxy-1-oxowitha-5,24-dienolide-27β-(*O*-β-d-glucopyranoside was also discovered latterly [53,54]. Furthermore, withacogulanoside-B along with five known withanolides was isolated [55]. The compounds present in different plant parts are given in Table 2. Furthermore, the structures of some withanolides are presented in Figure 2.

## 5. Application in the Food Industry

Berries of *W. coagulans* are well known for their milk coagulating potential. Keeping this property in view, the milk coagulating activity of protease from the plant was assessed. A temperature of 70 °C and pH 4 were found to be optimal for enzymatic activity. However, 60 °C was a stable temperature for the activity of the enzyme; SDS-PAGE showed a 66 kDa band [59]. Furthermore, the fruit extract of *W. coagulans* was utilized to assess the milk coagulating potential which demonstrated the highest impact at pH of 4 and temperature of 65 °C. Additionally, the time for rennet congealing of the extract was observed to be in direct relation with concentrations of NaCl or inversely with enzyme concentrations (protease inhibitors). Moreover, pepstatin-A (aspartic-protease inhibitor) completely inhibited the enzymatic potential of the berry extract [60].

An enzyme aspartic protease was isolated by using fractional ammonium-sulfate precipitation and cation-exchange chromatography from *W. coagulans* fruit. Furthermore, SDS-PAGE revealed the existence of a monomeric protein with a molecular weight of 31 kDa. The proteolytic activity of the protease enzyme was assessed using casein revealed K_m_ (1.29 mg/mL) and V_max_ (0.035 μmol Tyr/min) values for the protease enzyme. Skim milk was utilized to assess the milk coagulating potential of *W. coagulans* crude fruit extract. Consequently, mass spectrometry and inhibition assays revealed that aspartic protease is the only enzyme involved in milk coagulation. Additionally, the increasing salts concentrations (NaCl, CaCl_2_) gradually reduced the enzyme activity. Thus, it was concluded that this enzyme may be apt to produce the low salt cheese [61]. The protease was extracted from the berries of the plant and used for the production of white cheese. It was documented that cheese from *W. coagulans* was more acidic than cheeses prepared from other rennet sources [62].

Buffalo milk mozzarella cheese was developed by using fruits of *W. coagulans* as milk coagulants. Thus, an aqueous fraction of *W. coagulans* may be a suitable option for cheese production [63]. Buffalo milk cheese was developed by using an extract of the fruit of *W. coagulans* and was evaluated in terms of storage conditions (5 months). The highest content of ash, fat, crude protein as well as total solids was observed in cheese prepared with lyophilized berry extract [64]. Cheese preparation was done by using alcoholic and aqueous fractions of *W. coagulans* at different levels (0.5, 1, and 1.5%) containing plant proteinase [65].

Furthermore, the preparation of cottage cheese from an aqueous fraction of plant showed significantly higher moisture content as well as pH, however, no difference in ash, fat, and crude protein was observed in cheese prepared from calf rennet and *W. coagulans* [66]. An acceptable quality white cheese can be developed by the utilization of 0.5% alcoholic extract of the plant. The soy milk coagulating potential of *W. coagulans* extract was assessed in tofu preparation and compared with calcium-sulfate tofu. Sensory analysis revealed no difference between both types of tofu. However, yield as well as moisture content was lower in *W. coagulans*’s tofu [67].

## 6. Application in Nanotechnology

Silver nanoparticles (AgNPs) have prodigious potential on behalf of their mechanistic role in biomedical research. Approaches involving green chemistry have gained copious attention recently in plant science for the production of nanoparticles. Keeping in view this fact, leaf extract *W. coagulans* was utilized for the development of reduced graphene oxide (RGO)/Fe_3_O_4_ based nanocomposite with palladium nanoparticles (Pd/RGO/Fe_3_O_4_) and resulted in the reduction in 4-nitrophenol in the water at ambient temperature [68]. Silver nanoparticles were developed by using *W. coagulans* leaf extract and characterization of those nanoparticles was conducted by using UV–Vis, scanning electron microscopy, energy dispersive X-ray analysis, transmission electron microscopy, X-ray powder diffraction, and Fourier transform infra-red. The cumulative result indicated the size of particles as 14 nm having a spherical face-centered cubic structure [69].

Encapsulation of the water extract was performed by developing chitosan nanoparticles coated with food-based starch to retard extract release in the stomach. The release was retarded by 2.5 times by this method, hence exerting hypoglycemic potential [70]. *W. coagulans* was utilized to develop iron oxide nanorods. Iron oxide nanorods with an average size of 16 ± 2 nm and highly crystalline nature was obtained [6]. Furthermore, Keshari [71] also used *W. coagulans* extract to develop green silver nanoparticles. Those nanoparticles were crystalline, elemental, and spherical which also showed anti-biotic potential.

## 7. Therapeutic Potential of *W. coagulans*

**Figure 3:**
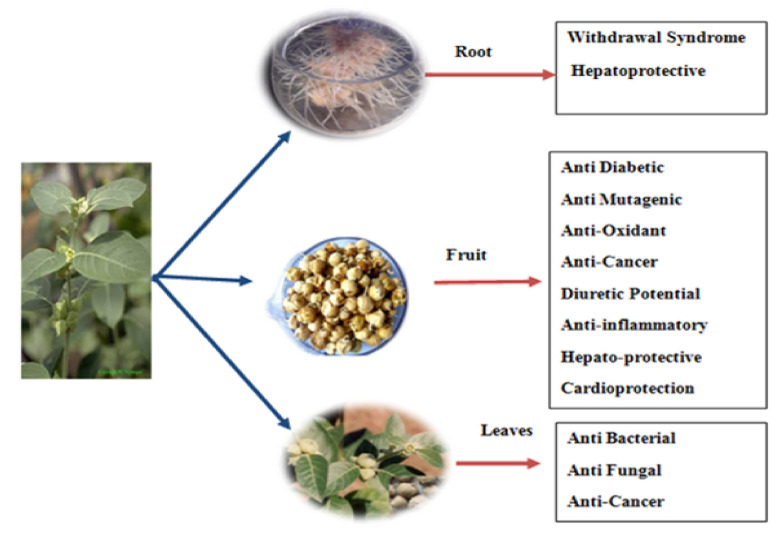
Graphical representation of the therapeutic potential of *W. coagulans* in several ailments.

**Figure 4:**
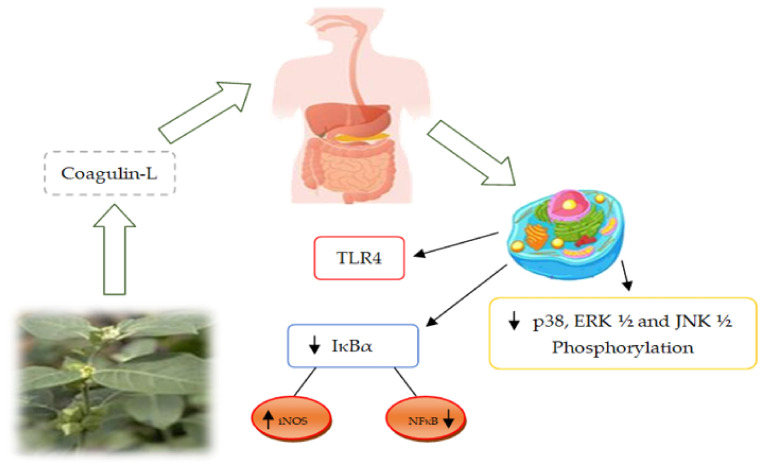
Anti-inflammatory potential of *W. coagulans* (IκBα: Nuclear factor of kappa light polypeptide gene, iNOS: Inducible NO synthase, NFκB: Nuclear factor of kappa light polypeptide gene, TLR4: Toll-like receptor 4, ERK1/2: Extracellular signal regulator kinase, JNK1/2: c-Jun-N-terminal kinase.

A number of medicinal properties are attributed to *W. coagulans* such as antifungal, anti-cytotoxic, antidiabetic, hypolipidemic, neuroprotective, anti-inflammatory, anti-cancerous, anthelmintic, antioxidant activity, and wound healing activity [15]. Various pharmacological and therapeutic activities of *W. coagulans* are attributed to the various plant parts including roots, leaves, and fruits as shown in Figure 3. The anti-inflammatory mechanism of action of cogulin L isolated from *W. coagulans* is illustrated in Figure 4. Available literature indicates the therapeutic role of *W. coagulans* and its withanolides is summarized in Table 3.

## 8. Conclusions

*W. coagulans* possesses considerable therapeutic potential and has been employed as a remedy against various disorders and diseases due to the presence of withanolides. It also possesses esterases, free amino acids, fatty oils, and essential oils. Medicinal properties of *W. coagulans* such as hepatoprotective, anti-inflammatory, hypoglycaemic, cardioprotective, free radical scavenging, antimicrobial, central nervous system depressant, immunomodulation, antitumor, and cytotoxic activities have been revealed by several pharmacological studies. However, future studies are needed to explore the mechanisms of action of compounds isolated from *W. coagulans* in higher animals to confirm their protective activity and safety. Crude extracts from different parts of the plant especially from the fruit have significant medicinal potential. Modern medications can be developed after thorough research into the mechanism of action, bioactivity, toxicity, and pharmacotherapeutic potential of plant-derived beneficial chemicals, as well as clinical trials and standardization.

## Figures and Tables

**Table 1:** Mineral composition of *W. coagulans* [4].

Minerals (mg/kg)	
**Macro-Minerals**	
Calcium	9260
Magnesium	35,280
Potassium	2450
Sodium	125
**Micro-Minerals**	
Iron	98.8
Copper	2.2
Zinc	40.2
Chromium	0.6
Cadmium	1.4
Lead	1.9
Nickel	1.8

**Table 2:** Important constituents of roots, aerial parts, leaves, and fruits of *W. coagulans.*

Molecules	Part of Plant	References
Withaferin A	Root	[35]
(20*R*,22*R*)-6α,7α-epoxy-5α,20-dihydroxy-1-oxo-witha-2,24-dienolide)	Root	[39]
(20*S*,22*R*)-6α,7α-epoxy-5α-hydroxy-1-oxo-witha-2,24-dienolide)		
Withacoagin: (20*R*,22*R*)-5α,20-dihydroxy-1-oxowitha-2,6,24-trienolide)		
Coagulin B, Coagulin C, Coagulin D, Coagulin E, Coagulin R	Aerial parts (leaves and stem), whole plant	[48,52]
Amyrin	Aerial parts (leaves and stem)	[50]
Withacoagulin A: (¼(20*S*,22*R*)-17β,20β-dihydroxy-1-oxowitha-3,5,14,24-tetraenolide)	Aerial parts (leaves and stem)	[56]
Withacoagulin B: (¼(20*R*,22*R*)-20β,27-dihydroxy-1-oxowitha-3,5,14,24-tetraenolide)		
Withacoagulin C: (¼(20*S*,22*R*)-14α,15α,17β,20β-tetrahydroxy-1-oxowitha-3,5,24-trienolide)		
Withacoagulin D: (¼(20*S*,22*R*)-14α,17β,20β,27-tetrahydroxy-1-oxowitha-3,5,24-trienolide)		
Withacoagulin E: (¼(20*R*,22*R*)-14β,20β-dihydroxy-1-oxowitha-2,5,24-trienolide)		
Withacoagulin F: (¼(20*R*,22*R*)-14β,20β-dihydroxy-1-oxowitha-3,5,24-trienolide)		
Withanolide L		
(22*R*)-14α,15α,17β,20β-tetrahydroxy-1-oxowitha-2,5,24-trien-26,22-olide)		
Coagulansin A: (14α,17*S*,20*S*,22*R*)-14,17,20,27-tetrahydroxy-1-oxowitha-2,5,24-trienolide) Coagulansin B: (3β,14α,20*S*,22*R*)-3,14,20-trihydroxy-1-oxowith-5-enolide) Withanolide P: (¼(17α,22*R*)-14,17,22-trihydroxy-1-oxoergosta-2,5,24-trien-26-oic acid δ-lactone)(14*R*,15*R*,17*S*,20*S*,22*R*)-14,15,17,20-tetrahydroxy-1-oxowitha-2,5,24-trienolide) (14*R*,15*R*,17*S*,20*S*,22*R*)-14,15,17,20-tetrahydroxy-1-oxowitha-3,5,24-trienolide) (14*S*,17*R*,20*S*,22*R*)-14,17,20-trihydroxy-1-oxowitha-2,5,24-trienolide) (14S,17*R*,20S,22*R*)-14,17,20-trihydroxy-1-oxowitha-3,5,24-trienolide)	Whole plant, aerial parts	[52,57]
(5,20α(*R*)-dihydroxy-6α,7α-epoxy-1-oxo-(5α)witha-2,24-dienolide)	Aerial part (leaves)	[36]
(Ergosta-5,25-diene-3β,24ξ-diol)	Fruit	[38]
(3β-hydroxy-2,3-dihydrowithanolide F)	Fruit	[37]
Withanolide D		
(3β,14α,20αF,27-tetrahydroxy-1-oxo-20*R*,22*R*-witha-5,24-dienolide)	Fruit	[58]
Withanolide H: (14α,20αF,27-trihydroxy-1-oxo-20*R*, 22*R*-witha-2,5,24-trienolide)		
Ajugin E	Fruit	[49]
Ajugin A	Fruit	[50]
Withacoagulin: (20β,27-dihydroxy-1-oxo-(22*R*)-witha-2,5,24-tetraenolide)	Fruit	[43]
(20β-hydroxy-1-oxo-(22*R*)–witha–2,5,24-trienolide)		
Coagulanolide (17*S*,20*S*,22*R*)-14α,15α,17β,20β-tetrahydroxy-1-oxowitha-2,5,24-trienolide)	Fruit	[51]
(20*R*,22*R*)-14,20α,27-trihydroxy-1-oxowitha-3,5,24-trienolide	Fruit	[56]

**Table 3:** Therapeutic potential of *W.* *coagulans.*

Part of Plant	Type of Intervention	Experimental Model	Dosage	Outcomes	References
**Cardioprotective Potential**					
Fruit	Methanolic extract	Rabbits (1–1.5 kg weight)	200 and 600 mg/kg BW	Improved lipid profile, HMG-COA reductase, lipase, and antioxidant activities	[9]
Fruit	Withacoagulin and coagulin C	Female Albino rats (100–120 g)	25 and 50 mg/kg BW	Antihypertensive impact in a dose-dependent manner	[72]
Fruit	Withacoagulin	Male Albino rats (120–150 g)	25 mg/kg BW	Superoxide dismutase, catalase, creatinine phosphokinase, and lactate dehydrogenase significantly reduced	[73]
**Hepatoprotective Activity**					
Fruit	Methanolic and aqueous-methanolic extracts (80%)	Albino rats (170–220 g)	800 mg/kg BW	An improvement as well as biosynthesis of liver and bile duct specific enzymes. Maintenance of the integrity of the hepatic membrane	[8]
**Anti-inflammatory and Immune Modulatory Activity**					
Fruit	Coagulin L	Human murine cells, mice model (male Swiss Albino mice)	1, 3, 10 μM (In vitro) 10, 25, and 50 mg/kg BW	Suppression of TLR4 induced immune-mediators including cytokines, growth factors, nitric and superoxide led towards immune-modulatory responses. Moreover, it reduced the degradation of IκBα which in turn inhibited the expression of NF-κB by downregulating the expression of iNOS and release of pro-inflammatory cytokines	[14]
Fruit	Ethanolic extract (50%)	Broiler chicken (550 male)	0, 75, and 150 mg/kg diet	The concentration of immunoglobulin G was significantly improved through improving humoral response at the dosage of 150 mg/kg diet	[13]
Aerial parts	Crude extract (methanol and chloroform in 1:1)	Sprague Dawley rats (180–220 g)	200, 100 and 50 mg/kg BW	Anti-inflammatory impact (70.0%)	[74]
Plant	Methanolic extract (80%)	Wistar rats (150–200 g)	250 and 500 mg/kg BW	Anti-inflammatory and antioxidant activity	[75]
Fruit	Aqueous extract	In vitro analysis	-	Strong antioxidant and free radical scavenging potential	[10]
**Antibacterial, Antifungal and Diuretic Activity**					
Roots and leaves	Chloroform, ethyl acetate, and aqueous extract	Bacterial strains (Gram-positive and Gram-negative)	0.5, 1, 1.5, and 2 mg/mL	Chloroform leaves and ethyl acetate stem extracts at a dose of 2 mg/mL had significant inhibition activity against bacterial pathogens as compared to aqueous extract	[76]
Fruit	Methanolic extract	*Klebsiella pneumonia*, *Escherichia coli*, *Salmonella paratyphi*, *Staphylococcus aureus*, *Bacillus subtilis*, and *Micrococcus luteus*	20 µg/mL	The highest inhibition by the methanolic extract was reported against *Bacillus subtilis* at 12 mm	[7]
Fruit	Methanolic extract	Male Charles Foster Albino rats (150–200 g)	400 mg/kg BW	The nephron-protective role was illustrated by the reduction in levels of free radical, renal function test, and protection from DNA damage	[77]
Leaves	Silver nanoparticles (leaf extract)	Bacterial strains (Gram-positive and Gram-negative)	5, 10, 15, and 20 µg/mL	It curbed the growth of both gram-positive and negative bacteria	[69]
Fruits	Silver nanoparticles (fruit extract)	*Enterococcus faecalis*, *Staphylococcus aureus*, *Escherichia coli*, *Proteus vulgaris*, *Salmonella typhi*, and *Vibrio cholera*	50 µg/mL	Phenolic constituents present in the *W. coagulans* can reduce silver nitrate into the silver nanoparticles. Moreover, bactericidal and bacteriostatic activity was elucidated	[71]
Fruit	Iron oxide nanorods (biological and chemical)	*Pseudomonasaeuroginosa* and *Staphylococcus* *aureus*	5, 10, and 20 µg/mL	The study indicated that biological nanorods are more effective (30% higher activity) than chemically prepared nanorods*. W. coagulans* nanoparticles showed significant inhibitory potential against *P. aeuroginosa* and *S. aureus* that indicates these nanoparticles are more effective than chemically prepared nanoparticles	[6]
Fruit	Aqueous extract	In vitro (silver carp fillet)	0.5% extract, 1% extract, 1% chitosan, 1% chitosan with 0.5% extract and 1% chitosan with 1% extract	Chitosan coating of extract demonstrated debility in levels of total bacterial counts and psychrophilic total bacterial counts as well as enhanced shelf life of fish fillets	[78]
**Hypoglycemic Potential**					
Whole plant	*n*-butanol and chloroform extract	In vitro and in silico	Ajugin E (66.7 ± 3.6 µM), withaperuvin C (407 ± 4.5 µM), withanolid J (683 ± 0.94 µM)	Withacogulanoside-B from *n*-butanol fraction and withaperuvin C as well as 27-hydroxywithanolide I with another 3 known withanolides (chloroform fraction) were identified. Among these, ajugin E showed higher α-glucosidase inhibition potential	[55]
Fruit	Ethanolic extract	Wistar rats and in vitro	400 mg/kg BW	*W. coagulans* suppressed the DPP-4 levels (63.2%) in an in vitro model at 14 μg/mL. Furthermore, restoration of pancreatic-endocrinal tissues was observed	[5]
Whole plant	Aqueous extract	Sprague Dawley rats	100 mg/kg BW	*W. coagulans* showed a promising impact on postprandial insulin level and amended the architecture of beta cells of the pancreas	[79]
Whole plant	Aqueous extract	Male Sprague Dawley rats (200–300 g)	1000 mg/kg BW	*W. coagulans* improved expression of glucagon-like peptide 1 which in turn reduced fasting as well as postprandial glucose levels	[80]
Fruit	Aqueous extract	In vitro (mice pancreatic β-cells) In vivo (Male ICR mice; 28–36 g)	In vitro (1, 2, 5, 10, and 25 μM) In vivo (50 mg/kg BW)	Secretions of insulin were promoted 2-fold in cells treated with the extract. Furthermore, in vivo testing corroborated to suppress the levels of blood glucose by 60%	[70]
Fruit	Aqueous extract	In vitro	0–100 µg/mL	Chromatographic analysis revealed the presence of 17β-hydroxywithanolide K, withanolide F, and coagulin C in fruit fraction that was further illustrated cytotoxic potential against HepG2 cells. Both EAF and WF promoted insulin secretions and inhibition of glucose absorption	[81]
Bud	Chloroform extract	In vitro (L6 rat skeletal muscle cells)	3.906, 7.8125, 15.62,5 31.25, 62.5, 125, 250 and 500 μg/mL	*W. coagulans* bud illustrated significant uptake of glucose via GLUT-4 and activity of PPAR gamma that resulted in enhanced glucose dumping and insulin sensitivity in skeletal muscles	[82]
**Anticancer Activity**					
Whole plant	Hydro-methanolic extract	Forty male Wistar rats (200–250 g)	1000 mg/kg BW	*W. coagulans* extract treatment induced cell apoptosis in the prostate and the expression of cyclooxygenase-2 in the prostatic tissues were effectively reduced	[83]
Fruit	Methanolic extract	Human breast cancer and normal kidney epithelial cell lines	20–200 μg/mL	Methanolic fruit extract showed substantial anticancer activity by reducing cell viability	[11]
Leaves	Methanol and chloroform extract	Cell cultures include normal and cancerous human prostate cell lines	10–250 μg/mL	Extract exerted its cancer-preventing action by inducing apoptosis, decreasing cell viability, invasion, cell proliferation, and migration of prostate cancerous cells	[84]
Whole plant	Water and methanol extract	Forty Wistar rats (200–250 g)	250, 500, and 1000 mg/kg BW	*W. coagulans* extract caused decreased malondialdehyde levels and increased total antioxidant capacity levels in the prostate gland	[85]
Fruit	Ethanol extract	Human breast cancer cell line	0, 10, 20, 40, 80, 160 and 320 µg/mL	Plant extract arrested cell cycle at G_2_/M phase and was found non-hemolytic	[86]
Root, leaf, leaf stalk, and fruit	Methanolic extracts	Human and rat cancer cell lines	20 μg/mL	The leaf stalk extract showed the highest cytotoxic activity against all tested cell lines	[87]
Leaf	Silver nanoparticles(leaf extract)	Cervical cancerous hyper-triploid cell-lines	0.25–30 mg/L	Silver nanoparticles containing withanolides unveiled cytotoxic and apoptotic potential	[69]
**Other Health Benefits**					
Whole plant	Hydroalcoholic extract	Male Wistar rats (48)	250, 500, and 1000 mg/kg BW/day	Results showed a significant decrease in sperm count, gonadosomatic index, and sperm viability	[85]
Whole plant	Ethanolic extracts	Vermicidal activity against *Pheretima posthuma* earthworm	75 and 100 mg/mL	*W. coagulans* extract exhibited remarkable anti-helminthic activity against *P. posthuma*	[17]
Fruit	Alcoholic extract	Swiss Albino mice	200, 500, and 1000 mg/kg BW	Fruit extract was evaluated as an antidepressant as it reduced the immobility and increased the mobility in rats through tail suspension test	[88]
Root	Ethanol water (3:1) extract	Male Wistar rats	500 and 1000 mg/kg BW	Neuro-protective potential against oxidative stress-induced injury was illustrated with enhancement in the number of intact neurons and suppression in the number of TUNEL neurons in the hippocampal region	[89]
Roots	Methanol and water (3:1)	Male Wistar rats (220–250 g)	1000 mg/kg BW	Preischemic extract administration effectively increased the antioxidant status (catalase, glutathione peroxidase, and superoxide dismutase level) and reduced the malondialdehyde level in the striatum brain region.	[90]
Whole plant	Methanol and chloroform (1:1).	Sprague-Dawley rats (180–220 g)	200, 100, and 50 mg/kg BW	The antinociceptive potential of *W. coagulans* estimated via hot plate assay elucidated pain reduction by 65.3% and 62% by writhing assay	[74]
Fruits	Methanolic extract	Mice	100 and 250 mg/kg BW	Study results suggested analgesic and sedative activity of *W. coagulans*	[91]
Fruit	Alcoholic extract	Swiss Albino mice	200 mg/kg, 500 mg/kg, and 1000 mg/kg	The alcoholic extract did not exhibit an antidepressant effect in rats, but it showed a depressive effect on mood	[92]
Fruit	Alcoholic extract	Swiss Albino mice	200, 500 and 1000 mg/kg BW	Rota road test also exhibited central nervous system depressant activity	[88]
Fruit	Alcoholic extract	Swiss Albino mice	200, and 1000 mg/kg BW	Results showed no considerable association between *W. coagulans* fruit extract and catalepsy	[93]
Fruit	Hydroalcoholic extract (50% ethanol)	Male one-day-old broiler chickens (600)	0, 100, or 200 mg/kg diet	Non-significant impact on the mineralization of tibia bone was illustrated. Dietary calcium level was declined by 30% and total antibodies level was not influenced significantly	[94,95]
Fruit	Hydroalcoholic extract	Male one-day-old broiler chickens (550)	150 and 75 mg/kg diet	*W. coagulans* and *W. somnifera* administration increased the bone mineralization	[96]

## Data Availability

Not applicable.
